# The Evolution of the *FT/TFL1* Genes in Amaranthaceae and Their Expression Patterns in the Course of Vegetative Growth and Flowering in *Chenopodium rubrum*

**DOI:** 10.1534/g3.116.028639

**Published:** 2016-07-28

**Authors:** Jana Drabešová, Lucie Černá, Helena Mašterová, Pavla Koloušková, Martin Potocký, Helena Štorchová

**Affiliations:** * Institute of Experimental Botany, Academy of Sciences of the Czech Republic, 165 02 Prague 6, Czech Republic; † Department of Experimental Plant Biology, Faculty of Natural Sciences, Charles University, 128 44 Prague 2, Czech Republic; ‡ Department of Genetics and Microbiology, Faculty of Natural Sciences, Charles University, 128 44 Prague 2, Czech Republic

**Keywords:** transcriptome, *FLOWERING LOCUS T/TERMINAL FLOWER1* gene family, evolution, flowering, gene rearrangement, Amaranthaceae, *Chenopodium rubrum*

## Abstract

The *FT/TFL1* gene family controls important aspects of plant development: *MFT*-like genes affect germination, *TFL1*-like genes act as floral inhibitors, and *FT*-like genes are floral activators. Gene duplications produced paralogs with modified functions required by the specific lifestyles of various angiosperm species. We constructed the transcriptome of the weedy annual plant *Chenopodium rubrum* and used it for the comprehensive search for the *FT/TFL1* genes. We analyzed their phylogenetic relationships across Amaranthaceae and all angiosperms. We discovered a very ancient phylogenetic clade of *FT* genes represented by the *CrFTL3* gene of *C. rubrum*. Another paralog *CrFTL2* showed an unusual structural rearrangement which might have contributed to the functional shift. We examined the transcription patterns of the *FT/TFL1* genes during the vegetative growth and floral transition in *C. rubrum* to get clues about their possible functions. All the genes except for the constitutively expressed *CrFTL2* gene, and the *CrFTL3* gene, which was transcribed only in seeds, exhibited organ-specific expression influenced by the specific light regime. The *CrFTL1* gene was confirmed as a single floral activator from the *FT/TFL1* family in *C. rubrum*. Its floral promoting activity may be counteracted by *CrTFL1*. *C. rubrum* emerges as an easily manipulated model for the study of floral induction in weedy fast-cycling plants lacking a juvenile phase.

Land plants evolved numerous adaptations to protect against drying, to ensure fertilization, and to cope with Earth’s gravity. The effective control of plant architecture was made possible by an intricate network of regulatory proteins, phytohormones, and growth substances (Vaňková *et al.* 2014). Novel gene families encoding transcription factors and cofactors appeared when plants conquered the land. One of them, the *FLOWERING LOCUS T/TERMINAL FLOWER1* (*FT/TFL1*) gene family, regulates the transition from the vegetative to the reproductive phase and controls plant growth (Wickland and Hanzawa 2015). The family can be divided into three phylogenetic clusters: *MOTHER OF FT AND TFL1* (*MFT*)-like, *TFL1*-like, and *FT*-like. The most ancestral clade includes *MFT*-like genes, found in mosses, lycopods, and all seed plants (Hedman *et al.* 2009). Their original function is related to growth arrest and dormancy, satisfying the essential need of land plants to control their size and architecture. The *TFL1*-like clade originated in seed plants in order to independently regulate seed and bud dormancy (Kalgren *et al.* 2011). The most recent *FT*-like clade arose with angiosperms. The FT-like proteins constitute the principal component of florigen, a long sought after substance promoting flowering (Corbesier *et al.* 2007; Jaeger and Wigge 2007). The evolution of the *FT/TFL1* genes was driven by extensive gene duplications. They occurred in all lineages of flowering plants and were often associated with functional shifts (Pin *et al.* 2010; Navarro *et al.* 2011; Wang *et al.* 2015).

Six members of the *FT/TFL1* gene family exist in *Arabidopsis thaliana*, a model long-day plant (Kardailsky *et al.* 1999; Kobayashi *et al.* 1999). The *FT* and *TWIN SISTER OF FT* (*TSF*) genes are floral promoters. In contrast with *FT*, the *TSF* gene is activated by cytokinins under nonpermissive short days (D’Aloia *et al.* 2011), demonstrating the functional diversification of both flowering activators. Members of the *TFL1* subfamily act as floral repressors. *TFL1* maintains inflorescence meristem in the shoot apex and delays its differentiation into floral organs (Simon *et al.* 1996). *ARABIDOPSIS THALIANA CENTRORADIALIS HOMOLOG* (*ATC*) functions as a graft transmissible floral repressor under short days (Huang *et al.* 2012). *BROTHER OF FT AND TFL1* (*BFT*) inhibits floral initiation under salt stress (Ryu *et al.* 2011). Finally, *MFT* is highly expressed in seeds and affects germination by interacting with abscisic acid and gibberellin signaling pathways (Xi *et al.* 2010).

Functional shifts of FT proteins have occurred in many plant species, *e.g.*, in sunflower (Blackman *et al.* 2010), tobacco (Harig *et al.* 2012), sugarcane (Coelho *et al.* 2014), and soybean (Wang *et al.* 2015). Novel FT-derived floral repressors carry mutations in the positions proven to be critical for regulatory function by site-specific mutagenesis in *A. thaliana* (Hanzawa *et al.* 2005; Ahn *et al.* 2006; Ho and Weigel 2014). For example, BvFT1 inhibits flowering in sugar beet, whereas its paralog BvFT2 is a floral promoter. This functional shift was associated with the exchange of Tyr-134 for Asn, and Trp-138 for Gln (Pin *et al.* 2010).

*Chenopodium rubrum* (red goosefoot) is the only representative of the family Amaranthaceae besides sugar beet, in which *FT*-like genes have been identified and their functions estimated in transgenic *A. thaliana*. The *CrFTL1* gene acted as a floral promoter similarly to its ortholog *BvFT2* in sugar beet (Pin *et al.* 2010). In contrast, the *CrFTL2* gene orthologous to the sugar beet repressor *BvFT1* was not involved in flowering control (Cháb *et al.* 2008; Drabešová *et al.* 2014). *C. rubrum* is a tetraploid species (Loeve and Loeve 1982), most likely an allotetraploid (Cháb *et al.* 2008; Kolano *et al.* 2012), which has originated quite recently according to its position on the phylogenetic tree (Fuentes-Bazan *et al.* 2012). Red goosefoot is a short-day annual without a juvenile phase. It represents an opportunistic weedy species which prefers disturbed, ephemeral habitats. It may be induced to flowering by a single period of darkness at the seedling stage (Cumming 1967). *C. rubrum* has long been used in classical physiological studies of flowering (Seidlová and Krekule 1973), and more recently in genetic studies (Veit *et al.* 2004; Cháb *et al.* 2008; Drabešová *et al.* 2014).

In the current work, we performed a comprehensive study of the *FT/TFL1* gene family in *C. rubrum*. We constructed a reference transcriptome, identified the members of this family, and generated the phylogenetic tree across angiosperms. The absence of the juvenile phase and the existence of only one floral activator from the *FT/TFL1* gene family makes *C. rubrum* an attractive model to study floral induction and for comparison with other amaranths.

## Materials and Methods

### Plant material and growth conditions

Seeds of *C. rubrum* ecotype 374 (Cumming 1967) were germinated in Petri dishes with wet filtration paper. Germinating seeds were planted in 96-well flat-bottom ELISA plates (one seedling per well). The wells were filled with perlite (particle size 0.4–0.8 mm) and perforated at the bottom (1 mm-hole) to provide a nutrient solution. The plates were floated in half-strength Hoagland solution which was replaced each second day. After 3 or 5 d, only average sized seedlings with opened cotyledons (75%) were maintained; the remaining plants were discarded. Selected plantlets were exposed to various photoperiodic treatments – permanent light, one or three periods of darkness followed by permanent light, and a diurnal regime with 12 hr light and 12 hr darkness (Supplemental Material, File S1). The modified floral dip method used in attempts to transform *C. rubrum* is described in File S2.

### RNA isolation

Leaf, root, stem, or apical tissues from seedlings, young, or mature plants cultivated under various photoperiodic regimens were collected and flash frozen in liquid nitrogen. Sampling in dark was performed under a dim green light (about 520 nm, Green Hornet LED headlight). Total RNA was isolated using a Plant RNeasy Mini kit (Quiagen, Valencia, CA). DNA contamination was removed by DNase I treatment according to the manufacturer’s protocol (DNA-free, Ambion, TX). RNA quality and concentration were checked on a 0.9% agarose gel and by NanoDrop (Thermo Fisher Scientific, Finland).

### RNAseq

We sequenced and assembled several *C. rubrum* transcriptomes, utilizing both 454 pyrosequencing and Illumina HiSeq to achieve a reliable reference transcriptome. The mixture of equal amounts of 44 RNA samples extracted from various tissues of seedlings or mature plants cultivated under various photoperiodic treatments in the growth chamber or in the greenhouse (200 μg in total) was dried in GenTegra tubes (GenTegra, Pleasanton, CA) and sent to Evrogen Joint Stock Company (Moscow, Russia) to prepare a normalized cDNA library (10 μg), which was afterward sequenced using Roche 454 GS-FLX platform with Titanium reagents in the DNA Core facility at Brigham Young University (Provo, UT). We obtained 2,285,349 raw reads from which 1,830,810 reads passed quality filtering. Median read length was 463 bp and the average was 412 bp. The total number of bases reached 754,859,592.

In addition, we selected RNAs isolated in two replicates from 9 organs and developmental stages and sent 12 samples to the University of Southern California Epigenome Center (CA) for library preparation and HiSeq Illumina sequencing, which generated paired end reads (50 bp, fragment size about 200 bp).

### Transcriptome assembly

Raw Illumina reads were processed to eliminate artifacts from library construction and low quality reads with the Trimmomatic v0.32 software (Bolger *et al.* 2014). We trimmed all edge bases below quality score 20 and discarded reads shorter than 48 bp. Orphan reads were assigned as single reads. Only paired end reads were used for the reference transcriptome assembly.

454 reads were assembled using Newbler (Gs *De novo* Assembler v2.8, Roche, Germany) with default settings. A file with the list of sequencing adaptors and primers was specified in order to remove sequencing and PCR artifacts.

For Illumina data we used the following four *de novo* assemblers: Trinity v2013-02-25, v2014-04-13, and v2.0.6 (Grabherr *et al.* 2011) SOAPdenovo-Trans v1.03 (Xie *et al.* 2014), Velvet-Oases v0.2.09 (Schulz *et al.* 2012), and Trans-Abyss v1.5.1 (Robertson *et al.* 2010). Trinity was used with the default k-mer size 25, SOAPdenovo-Trans was used with k-mer sizes 20−45 and a step size of 5, Velvet-Oases was used with k-mer sizes 21−29 and step size of 2, and Trans-Abyss was used with k-mer sizes 25−45 and a step size of 2. The assemblies calculated with various k-mers by the same assembler were combined to produce a merged assembly. The utilities offered by individual software packages were applied. Raw data were deposited in SRA database under the accession number PRJNA305086.

Transcriptome assemblies were performed with a 12-core Linux workstation with 60 GB memory (Laboratory of Plant Reproduction, IEB) and with computing resources of virtual organization Metacentrum (Czech National Grid Organization). Contigs shorter than 200 bp were discarded from all assemblies. Redundant contigs were further discarded using CD-HIT v4.6 (Li and Godzik 2006) with settings -c 1.0 -n 5. Contigs shorter than 200 bp were excluded from assemblies and the “best” set of assembled transcripts were than selected using the tr2aacds pipeline from the EvidentialGene package v2013.07.27 (http://arthropods.eugenes.org/about/about-EvidentialGene/EvidentialGene_trassembly_pipe.html). Only the primary (main) output transcripts were used for follow-up analyses. The coverage of selected genes was quantified using RSEM (Li and Dewey 2011) bundled with the Trinity software package. Reads were aligned to the reference transcriptome using the Bowtie aligner (Langmead *et al.* 2009). Coverage values were *in silico* normalized as FPKM (Fragments Per Kilobase of target transcript length per Million reads mapped) (Trapnell *et al.* 2010).

### Assembly quality metrics

Several tools were adopted to compare the quality of the assemblies. The first method estimated the proportion of completeness of each assembled transcript by the Ortholog Hit Ratio (OHR) (O’Neil *et al.* 2010), where an OHR close to 1.0 indicates that the particular transcript was assembled to its full length. The transcripts were aligned against two reference databases: 357 ultraconserved ortholog coding sequences from *A. thaliana* (Kozik *et al.* 2008), and a list of 959 single copy nuclear genes shared among *Arabidopsis*, *Oryza*, *Populus*, and *Vitis* (Duarte *et al.* 2010). Whereas OHR refers to the completeness of the newly assembled transcripts, the following two metrics—contiguity and completeness (Martin and Wang 2011)—consider the coverages of selected reference transcripts. Completeness is defined as the percentage of expressed reference transcripts covered by all the assembled transcripts, contiguity is the percentage of expressed reference transcripts covered by a single, longest-assembled transcript. The threshold value is the proportion of a particular reference transcript that is covered by an assembled transcript. Both metrics were calculated according to Zhang *et al.* (2013). Open reading frames in assembled transcripts were identified and translated using the protein databases of five species: *Citrus clementina*, *C. sinensis*, *Vitis vinifera* (downloaded from the Phytozome, Goodstein *et al.* 2012), *Beta vulgaris* (downloaded from the *Beta vulgaris* Resource: bvseq.molgen.mpg.de, Dohm *et al.* 2014), and *Arabidopsis thaliana* (downloaded from the TAIR10, www.arabidopsis.org). Protein sequences were then aligned against databases used also for the calculations of OHR as described above (Kozik *et al.* 2008; Duarte *et al.* 2010).

RSEM-EVAL package, part of DETONATE (*De novo* TranscriptOme rNa-seq Assembly with or without the Truth Evaluation) toolkit was used to calculate each assembly score (Li *et al.* 2014). Due to a big number of contigs in the Oases assembly we were not able to calculate the score for this assembly. Chimeric transcripts were identified and cut using the approach described by Yang and Smith (2013). Chimera identification was based on *blastx* analyses against protein databases derived from five different species as described above.

### RT qPCR

One microgram of RNA and oligo dT primers (500 ng) were heated for 5 min at 65°, chilled on ice, and mixed with Transcriptor buffer (Roche, Mannheim, Germany), 0.5 μl of Protector RNase Inhibitor (Roche, Germany), 2 μl of 10 mM dNTPs, and 10 units of Transcriptor Reverse Transcriptase (Roche, Germany). The first strand of cDNA was synthesized at 55° for 30 min. RNA samples were reverse transcribed in two independent RT reactions and each cDNA specimen was measured twice.

The first strand of cDNA was diluted 10−20 times and qPCR was performed using the LightCycler 480 SYBR Green I Master (Roche, Germany) in a final volume of 10 μl with 300−500 nM of each of the high performance liquid chromatography (HPLC) purified primers (Table S1), supplied by Metabion (Germany). The LightCycler LC 480 (Roche, Germany) was programmed as follows: 10 min of initial denaturation at 95°, then 40 cycles for 10 s at 95°, 10 s at 60° (*CrTFL1*, *CrBFT*, *CrMFT1*, *CrMFT2*, *CrCAB*) or 8 s at 58° (*actin*, *CrFTL1*, *CrFTL2*), followed by 15 s at 72°. PCR efficiencies were estimated from calibration curves generated from serial dilution of cDNAs. A calibrator was used to correct for run-to-run variation. The relative ratio of the target and reference gene was calculated as follows:

$${\text{E}}_{\text{R}}^{{\text{C}}_{\text{p}}\text{R}}/{\text{E}}_{\text{T}}^{{\text{C}}_{\text{p}}\text{T}}$$

where E_T_ /E_R_ represents the efficiency of target/reference amplification and C_p_T/C_p_R represents the cycle number at target/reference detection threshold (crossing point). Expression values were normalized with *actin* (Drabešová *et al.* 2014). This reference gene could not be applied to RNA extracted from seeds, because of its low expression. Normalization by cDNA (Libus and Štorchová 2006), followed by the recalculation with regard to *actin*-normalized samples, was therefore adopted for RNA extracted from dry or imbibed seeds. The specificity of qPCR was confirmed by amplicon resequencing.

### Identification of the FT/TFL1 genes

A local database was made from the reference transcriptome of *C. rubrum*. It was searched using the *tblastn* algorithm with *A. thaliana FT/TFL1* protein sequences as queries. The first hits were blasted against an *A. thaliana* protein database using *blastx* to confirm mutual homology. The coding sequences of the *FT/TFL1* genes in *C. rubrum* were amplified by PCR with primers (Table S1) designed according to sequences from the reference transcriptome and with cDNA as a template. Another set of primers (Figure S1 and Table S1) was developed to amplify the *CrFTL* genes from genomic DNA. Cleaned PCR products (GeneJET PCR purification kit, Thermo Fisher Scientific, Finland) were sent to Macrogen Europe Laboratory (Netherlands) for Sanger sequencing. All GenBank accession numbers including newly obtained sequences and the list of species are given in Table S2.

Spinach *FT/TFL1* genes were identified in the Spinach 1.0.1 genomic draft published on http://bvseq.molgen.mpg.de/blast/ using the *tblastn* tool with *C. rubrum* queries and named according to their respective *A. thaliana* or *C. rubrum* homologs. The exons recognized in genomic sequences according to their similarity with *C. rubrum* genes were joined together to provide virtual sequences of the particular transcripts. Two copies of the *SoFTL1-2* gene found in close mutual vicinity in the scaffold 8759 were designated *SoFTL1-2a* and *SoFTL1-2b*. The *SoTFL1* gene was assembled from the sequences found in two scaffolds, 68,707 and 48,765, most likely due to a misassembly of the spinach genomic draft. Sugar beet genes were retrieved from GenBank by *blastp* with *C. rubrum* protein queries, except for *BvFTL3*, which was identified in RefBeet-1.1 (http://bvseq.molgen.mpg.de/blast/). Owing to an inherent incompleteness of transcriptomes, Amaranthaceae transcriptomes published by One Thousand Plants (1KP) Consortium were not used for data mining.

### Phylogenetic analyses

Multiple nucleotide sequence alignment of coding regions guided by the protein sequence alignment was conducted using MUSCLE (Edgar 2004) with default parameters, as implemented in Geneious 7.1.5, and extensive manual editing. The alignment was analyzed by the maximum-likelihood (ML) method using RAxML (Stamatakis 2014). A γ model of rate heterogeneity was applied on three distinct data partitions corresponding to three codon positions. Bootstrap support of the majority rule consensus tree was calculated from 1000 pseudoreplicates. Gaps were treated as missing characters. The proportion of synonymous (Ks) and nonsynonymous (Ka) substitutions between two sequences was calculated using DnaSP v.5 (Librado and Rozas 2009).

### 3D structure model

Amino acid CrFTL2 sequence in FASTA format was uploaded through the I-TASSER web interface (http://zhanglab.ccmb.med.umich.edu/I-TASSER/) together with the template FT (*A*. *thaliana*, MMDB ID: 33642) in Program Database (PDB) format. In addition, a 3D model of CrFTL2 was independently computed with Modeler sofware (Webb and Sali 2014) using multiple templates (pdb codes 1wkp, 3axy, 1qou, 2iqy, 2jyz) in order to avoid biases introduced by single template and prediction algorithm.

### Data availability

The authors state that all data necessary for confirming the conclusions presented in the article are represented fully within the article.

## Results

### Transcriptome assembly

454 pyrosequencing produced 2,285,349 raw reads from which 1,830,810 reads passed quality filtering. We also obtained ∼25 million raw reads from each of 12 RNA samples sequenced by Illumina (altogether 293,391,190 raw read pairs). 253,682,808 read pairs (86.5%) passed quality check and were used for assembly. A single assembly was generated from the 454 data set by Newbler and 22 assemblies were created from all Illumina reads by four distinct assemblers. A combined superassembly was produced by the EvidentialGene pipeline. The assembly characteristics are summarized in Table 1.

**Table 1:** The characteristics of *C. rubrum* transcriptome assemblies produced by various assemblers

	Contigs				Chimeras		DETONATE	Contiguity 80%		Completeness 80%	
Assembler	Total Number	Average Length	Nonredundant	N50	Transcripts	%	Score	*Beta vulgaris*	*Arabidopsis*	*Beta vulgaris*	*Arabidopsis*
Trinity v2013-02-25	176,176	1165	176,176	2240	4364	2.48	−13300254357	0.839	0.897	0.804	0.855
Trinity v2014-04-13	109,230	1224	109,230	2130	1661	1.52	−11942708245	0.888	0.849	0.914	0.873
Trinity v2.06	103,364	839	103,272	1388	606	0.59	−13489216000	0.857	0.815	0,915	0.875
Trans-Abyss (MK)	478,002	687	477,947	1041	2028	0.42	−10478540027	0.917	0.882	0.937	0.897
Velvet-Oases (MK)	1,025,841	1022	681,326	1788	11,337	1.66	/^*a*^	0.869	0.823	0.938	0.904
SOAPdenovo-Trans (MK)	698,830	163	692,919	300	145	0.02	−20560646452	0.306	0.251	0.926	0.825
Newbler	22,587	1227	22,580	1354	14	0.06	−16700290718	0.657	0.615	0.687	0.643
EvidentialGene	40,487	912	40,487	1383	24	0.06	−15661125094	0.885	0.846	0.894	0.856

a Not calculated.

Newbler assembled reads derived from the normalized cDNA library. It produced 22,587 contigs (N50 = 1354) with nearly zero redundancy. Trans-Abyss generated fewer transcripts (478,002) than Oases or SOAPdenovo-Trans, N50 = 1041. The newest version of Trinity (2015) was the best in terms of a low proportion of chimeric transcripts and zero redundancy. Finally, a combined superassembly was compiled by the EvidentialGene pipeline from all the assemblies except of those produced by SOAPdenovo-Trans. The main output comprised 40,487 transcripts with no redundancy, defined as 100% nucleotide sequence identity. Basic assembly characteristics (for example N50) did not allow to decide which assembly should be chosen as a reference transcriptome. We have therefore employed additional quality metrics to evaluate the assemblies.

### Assembly quality metrics

The highest completeness (93.7%) and contiguity (91.7%) values, estimated using *Beta vulgaris* reference, were achieved by Trans-Abyss (Table 1). Using *A. thaliana* as a reference brought consistent results with slightly lower values owing to a large phylogenetic distance between *Chenopodium* and *Arabidopsis*. Trans-Abyss was immediately followed by Trinity and Oases. The assembly of the normalized cDNA library performed by Newbler exhibited only moderate completeness (68.7%) and contiguity (65.7%). Newbler used a 454 data set, which contained <1% of reads compared to the Illumina data set, though the reads were much longer. The worst performing program was SOAPdenovo-Trans with the lowest contiguity: 30.6%. This ranking was confirmed by DETONATE metrics.

The EvidentialGene superassembly (main output) showed the second highest contiguity (88.5%), a slightly lower completeness (89.4%) and a much lower number of transcripts (40,487). The reduction in transcript number was achieved owing to decreased redundancy and chimerism (Table 1), which represent a challenge for the assembly of the transcriptomes in polyploid plants (Nakasugi *et al.* 2014) Thus, we selected this superassembly as a suitable reference transcriptome of. *C. rubrum* for further studies. At the same time, we retained the Newbler, Trans-Abyss, and Trinity assemblies, and the alternative set of contigs from EvidentialGene superassembly as useful resources to search for rare or alternative transcripts.

### Evolution of the FT/TFL1 genes in Amaranthaceae

We identified eight members of the *FT/TFL1* gene family in the reference *C. rubrum* transcriptome, including the previously described *CrFTL1* and *CrFTL2* genes (Cháb *et al.* 2008). Most transcripts occurred in two slightly different variants (nucleotide sequence similarity of coding regions >98%) most likely derived from two homeologous copies existing in tetraploid *C. rubrum*. Owing to a very low divergence of the homeologous pairs, only one representative sequence (confirmed by Sanger sequencing) was included in phylogenetic analyses. Only three short reads transcribed from the *CrFTL3* gene were found in all data sets. Thus, the entire C*rFTL3* genomic sequence was obtained by Sanger sequencing genomic DNA (Figure S1) and the *CrFTL3* coding region was predicted from it.

**Figure 1:**
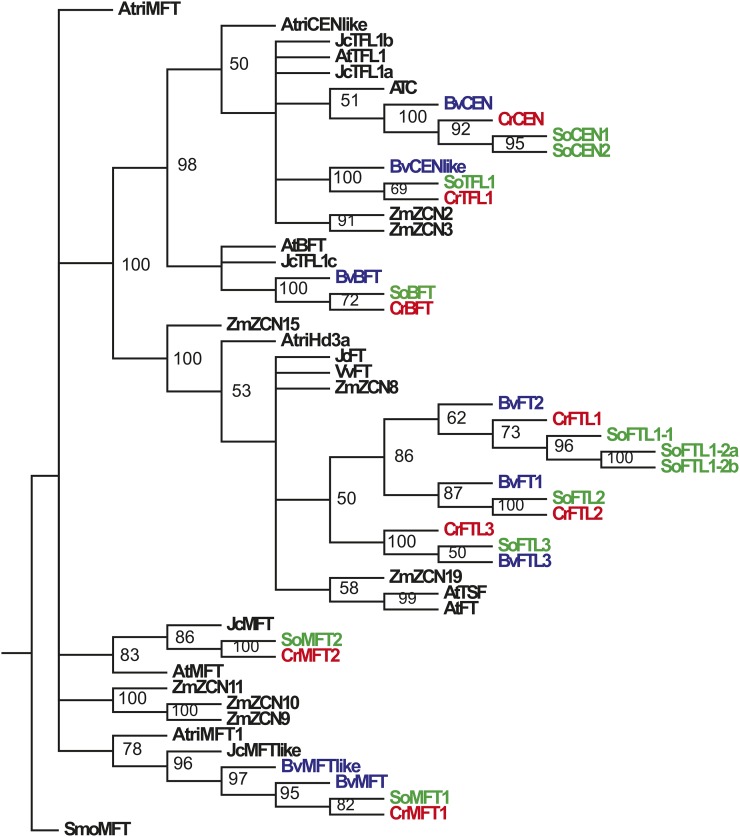
The ML phylogenetic tree of the *FT*/*TFL1* genes in angiosperms constructed by RAxML. Bootstrap support of the majority rule consensus tree was calculated from 1000 pseudoreplicates. GenBank accession numbers are given in Table S2. Species abbreviations: Cr, *Chenopodium rubrum* (red); Bv, *Beta vulgaris* (blue); So, *Spinacia oleracea* (green); At, *Arabidopsis thaliana*; Atri, *Amborella trichopoda*; Jc, *Jatropha curcas*; Smo, *Selaginella moellendorffii*; Zm, *Zea mays*. ATC, *CENTRORADIALIS* (*A. thaliana*).

We aligned the *FT/TFL1* homologs from *C. rubrum*, spinach, and sugar beet together with *Amborella trichopoda*, *Zea mays*, *Arabidopsis thaliana*, and *Jatropha curcas* genes and constructed a ML phylogenetic tree depicting the evolutionary history of the *FT/TFL1* family in angiosperms (Figure 1). The phylogram shows two well-supported clades corresponding to the *FT* and *TFL1* subfamilies. Furthermore, it contains unresolved basal branches representing the members of the *MFT* subfamily–the single *AtriMFT* gene from *A. trichopoda*, the cluster of *Z. mays* genes, and two clades with *C. rubrum MFT* paralogs. The *CrMFT1* gene clustered together with spinach *SoMFT1* and two *BvMFT* paralogs. The *CrMFT2* gene and its spinach homolog belonged to the same clade as *A. thaliana MFT*.

The gene duplication in *C. rubrum* and sugar beet, which generated *CrFTL1* and *CrFTL2* (Cháb *et al.* 2008), and *BvFT2* and *BvFT1* (Pin *et al.* 2010), was well documented in the *FT* clade. Moreover, another cluster composed of *FTL3* genes diverged before this duplication, but after the separation of Amaranthaceae genes from monocots and *Vitis vinifera*. We found no other *FTL3* homologs in any public database except for spinach and sugar beet. One homeolog pair of each of the *BFT*, *CEN*, and *TFL1* genes was identified in *C. rubrum*. The *BFT* genes formed the clade at the base of the *TFL1* subfamily.

The evolution of the *FT/TFL1* genes was accompanied by gene duplications and gene losses. The deepest gene duplication occurred in the *MFT* lineage in early angiosperm evolution. *C. rubrum* and spinach, as well as *J. curcas* retained both paralogs, but *MFT2* was lost in sugar beet. Instead, two *MFT1* copies evolved in sugar beet. The *FT* clade exhibited the duplication leading to *FTL2* genes, but also a more ancient event creating the *FTL3* cluster. Recent duplications of *SoCEN* and *SoFTL1* genes occurred in spinach.

### Genomic structure of the CrFTL2 gene and 3D structure of the CrFTL2 protein

**Figure 2:**
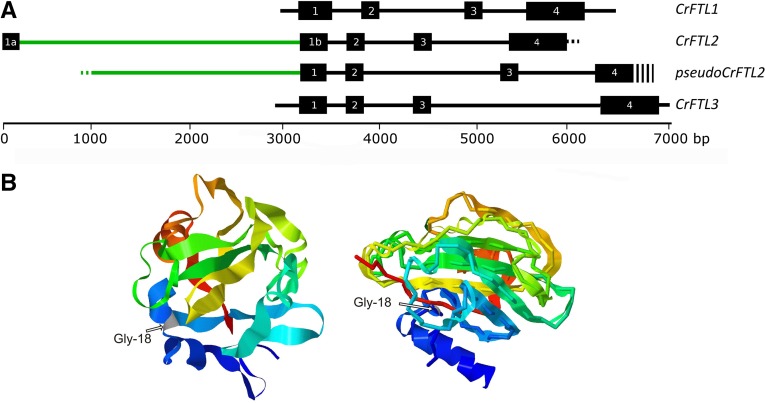
(A) The genomic structures of the *CrFTL* genes in *C. rubrum*: *CrFTL1*, *CrFTL2* with the additional intron and the novel exon 1a, *CrFTL2* pseudogene, *CrFTL3*. (B) The 3D structure of the CrFTL2 protein as predicted by Modeler (left) or I-TASSER using an *A. thaliana* FT template (right). The CrFTL2 chain is depicted as a ribbon with a blue N-terminus and a red C-terminus. The FT chain is represented by a backbone of the same color. Arrows show Gly-18, the starting point of the sequence homology between FT and CrFTL2.

To understand the structural evolution of the *CrFTL* genes, we determined their genomic sequences (Figure S1). We revealed a large intron (3157 bp) inserted in the first exon of the *CrFTL2* gene. Unlike the other angiosperm *FT*/*TFL1* genes which contain three introns and four exons, *CrFTL2* harbors four introns and five exons. The novel exon 1a replaced part of the former first exon in a mature transcript and coded for a stretch of 17 amino acids with no significant sequence similarity to anything. A fragment of a novel intron (about 1100 bp) was highly similar (80%) to the first exon of the *CHOLINE TRANSPORTER LIKE* gene (*CTL*) from sugar beet (XM_010692082). However, this intron was spliced out leaving no *CTL* sequence in the mature *CrFTL2* transcript. The substantial rearrangement of the *CrFTL2* gene was most likely achieved by recombination which removed the start of the first exon and replaced it with an additional, long intron and a novel exon (Figure 2A).

We constructed 3D models of the CrFTL2 protein using both multitemplate modeling and *A. thaliana* FT only as a template (Figure 2A). The two 3D predictions were very similar and showed an α helical structure at the N-terminus similar to FT protein and animal phosphatidylethanolamine-binding proteins (Ahn *et al.* 2006). Thus, the novel exon encoded a very similar 3D structure as the original one despite a completely different primary sequence. The CrFTL2 protein lacked 11 amino acid residues at the C-end, which might have changed its ligand-binding capacity.

We also identified a homeolog *CrFTL2* copy carrying the mutation in the splice site (AG > TG) upstream of the fourth exon, which prevented the production of a functional transcript. This copy also lacked exon 1a. Thus, only one of the *CrFTL2* homeologs was functional, the other one was a pseudogene. The *CrFTL1* and *CrFTL3* genes harbored a typical gene structure with variable intron sizes (Table S3).

### Amino acid divergence in the FT subfamily in Amaranthaceae

**Figure 3:**
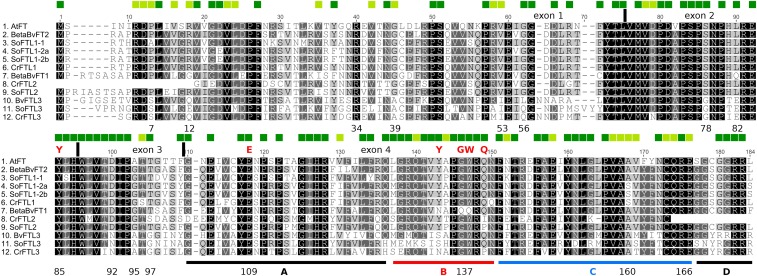
The alignment of the FTL proteins from *A. thaliana*, sugar beet, spinach, and *C. rubrum*. The squares above the alignment mark the residues conserved in >95% (darker) or >90% angiosperm FTL proteins according to Ho and Weigel (2014). The exons and the A, B, C, D segments of the fourth exons are designated. The amino acids essential for the floral promoting function are shown above the alignment. The amino acid positions in CrFTL3 important for the comparison with other FTLs are given under the alignment. The sequence derived from the first exon unrelated to other FTL proteins was excluded in CrFTL2.

We aligned amino acid sequences of the FT subfamily from sugar beet, spinach, and *C. rubrum* with *A. thaliana* FT protein (Figure 3). The FTL1 proteins of the three amaranths showed identity in almost all conserved positions (marked dark green in Figure 3) and high mutual sequence similarity (83–93%). The FTL2 proteins carried mutations in the positions critical for a regulatory function (Ho and Weigel 2014). Gln-140 was exchanged for Pro in SoFTL2, or for Ile in CrFTL2. Trp-138 was replaced by Gln in BvFT1, which turned the floral activator into a repressor in sugar beet (Pin *et al.* 2010). The CrFTL2 protein diverged very much from its counterparts, exhibiting only 78% similarity with its closest homolog in spinach. In addition to the different N-terminus encoded by the novel exon 1a, it lacked the conserved GGRR motif at the C-terminus owing to a premature stop codon. The Ka/Ks ratio (0.41) calculated from the nt alignment of *CrFTL2* and *SoFTL2* suggested relaxed selection constraints. This finding together with the constitutive expression of the *CrFTL2* gene (Cháb *et al.* 2008), not compatible with a floral repressor function, indicates subfunctionalization.

The FTL3 lineage belonged to the FT subfamily based on the phylogenetic tree and also on the occurrence of critical amino acid residues. Tyr-85, Glu-109, Trp-138, Gln-140, and Asn-152 (Figure 3) were all conserved with Pro-140 in CrFTL3 a notable exception. In contrast, FTL3 proteins contained unique amino acid residues in the positions conserved in other FT/TFL1 proteins: Ser-7, Ala-39, Gly-78, Thr-163 (Table S4). Synapomorphic amino acid replacements in the FTL3 lineage indicated possible changes of function.

### Plant growth under various light regimes

**Figure 4:**
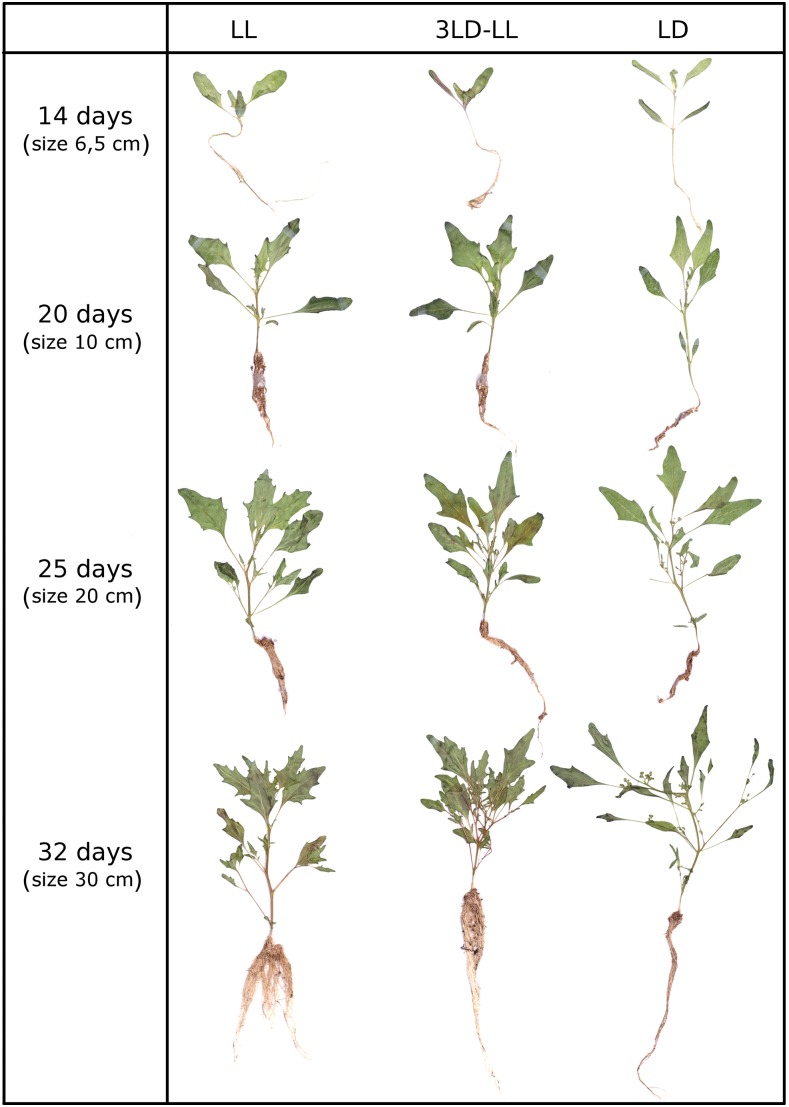
Phenotypes of *C. rubrum* plantlets growing under three light regimes. LL: permanent light; 3 LD–LL: three consecutive periods 12 hr light/12 hr dark followed by permanent light; LD: 12 hr light/12 hr dark. The actual size (in cm) is given on the left. The plants were pressed and scanned.

We grew *C. rubrum* plants till the age of 32 d under three distinct light regimes: (1) constant light (LL), (2) 12 hr D–12 hr L during an entire experiment (LD), and (3) three periods 12 hr D–12 hr L applied to 5-d-old seedlings followed by permanent light (3LD LL) (File S1). The first regime did not induce flowering, whereas the second and third one resulted in flowering in all plants. Constant light used as a control regime suppressing flowering may be considered to be a nonphysiological treatment. However, the *C. rubrum* ecotype 374 was collected close to the Arctic Circle (Cumming 1967). Constant light represents natural conditions in its native sites in the weeks around the summer solstice. We collected the plants and prepared herbarium specimens to document their habitus (Figure 4). Phenotypes of LL and 3LD LL plants were similar which documented a strong morphogenetic effect of constant light. Seedlings treated with at least three dark periods produced longer hypocotyls and roots that were twice as short as those in LL plants.

Mature (25 d) LD plants were branched with well visible inflorescences. In contrast, LL and 3LD LL plants were shorter and less branched and produced broader leaves. Their root systems were dense, with many adventitious roots, in contrast with the narrow root systems of LD plants (Figure 4).

### Organ-specific expression of the FT/TFL1 genes

**Figure 5:**
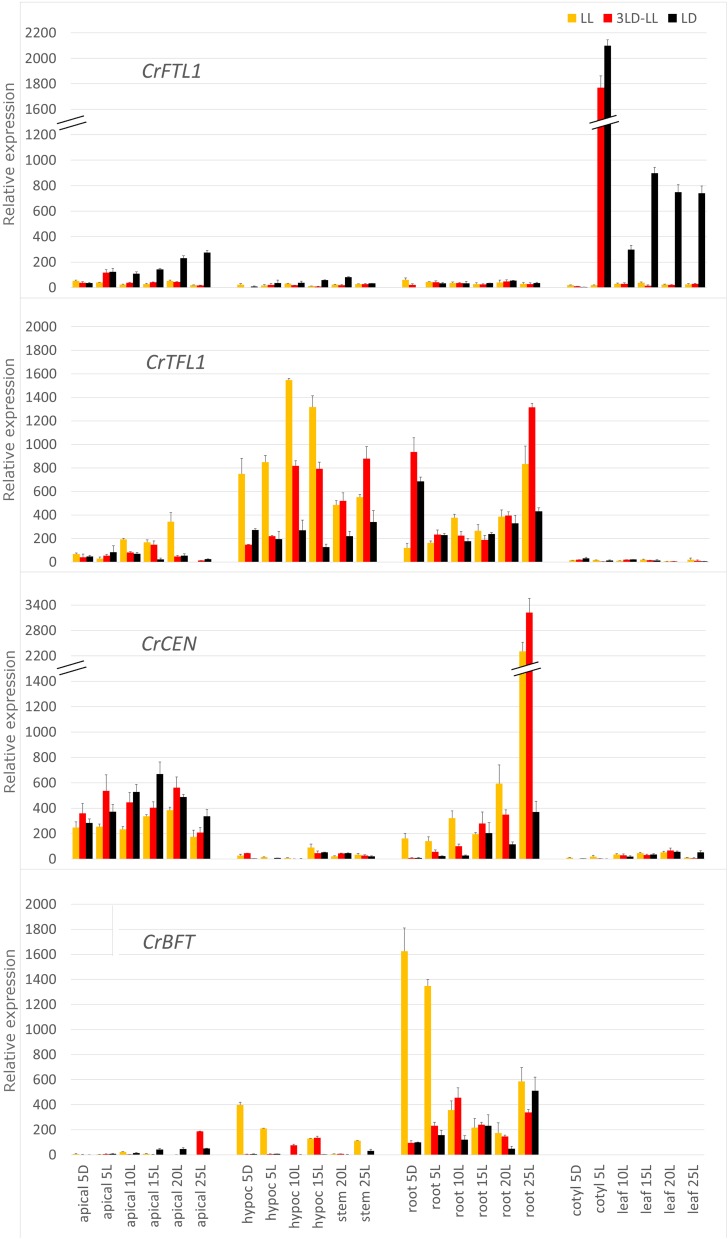
Relative expression of the *CrFTL1*, *CrTFL1*, *CrCEN*, and *CrBFT* genes in apical parts, hypocotyls, cotyledons, roots, stems, and leaves of *C. rubrum* plants under three light regimes. LL: permanent light (yellow); 3 LD–LL: three consecutive periods 12 hr light/12 hr dark followed by permanent light (red); LD: 12 hr light/12 hr dark (black).

The expression of the *TFL1* subfamily in whole aerial parts of seedlings was very low. Only *CrTFL1* transcripts were measurable; they displayed a weak diurnal rhythmicity under both short-day and long-day conditions (Figure S2 and File S3). However, when gene expression was estimated in separated organs, prominent transcript patterns were discovered (Figure 5).

We followed transcripts of the *FT*/*TFL1* genes by RT qPCR in roots, hypocotyls, cotyledons, and apical parts of *C. rubrum* seedlings, as well as in stems, roots, leaves, and apical parts of mature plants cultivated under three light regimes as described above (Figure 5). The first RNA collection occurred in the middle of the third dark period applied on light-grown seedlings 5 d old. The second sample was taken in the middle of the following light period. Seedlings grown under permanent light were sampled at the same time. We continued RNA collection in 10 d-, 15 d-, 20 d-, and 25-d-old plants, always in the middle of a light period.

High activation of the floral promoter *CrFTL1* was observed in cotyledons treated by the inductive dark period (Figure 5). Increased *CrFTL1* expression was recorded in leaves of mature LD plants, albeit at a lower level than in cotyledons, and also in LD apical parts. The *CrTFL1* transcription profile was complementary to *CrFTL1* in terms of organ specificity. High expression was observed in hypocotyl, stems, and roots, but was zero in leaves. The *CrTFL1* transcript levels in aerial parts were higher in constant light. It was inhibited at night and also during the day following the dark period. The *CrTFL1* transcription remained low in LD plants, but increased when 3LD LL plants were transferred to permanent light. Darkness activated *CrTFL1* expression in roots, opposite to the effect in hypocotyls. *CrTFL1* expression remained low when root samples were taken at light under any regime.

The *CrCEN1* gene was moderately expressed in apical parts with only small differences among the light regimes. Its expression increased in roots of mature plants grown at light. In contrast, the *CrBFT* gene was highly expressed in roots of seedlings grown under constant light and its transcript levels dropped in mature plants. *CrBFT* expression in light-grown hypocotyls might be an overflow of root expression (Figure 5). In general, the members of the *TFL1* subfamily substantially differed in expression patterns and also in their responses to light regimes.

The *CrMFT1* gene was transcribed in roots. It seemed to be activated by light in roots and also in stems of 25-d-old plants. *CrMFT2* expression was low in all organs except for a single outlier: the roots of 25-d-old plants (Figure S3).

**Figure 6:**
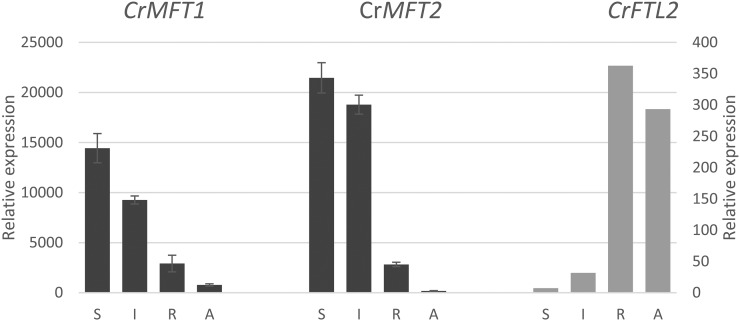
Relative expression of the *CrMFT1*, *CrMFT2*, and *CrFTL2* genes in seeds and germinating seedlings of *C. rubrum*. Dry seeds (S), imbibed seeds (I), primary roots (R), and aerial parts of seedlings before cotyledon opening (A). The left y axis expression values refer to *CrMFT* genes, the right y axis refers to *CrFTL2*.

In addition to vegetative tissues, we also examined gene expression in *C. rubrum* seeds (Figure 6). The two *CrMFT* paralogs exhibited very high expression in dry and imbibed seeds, with a sharp decrease following germination. Other *FT*/*TFL1* genes, including *CrFTL2*, showed zero or very low transcript levels in seeds. Interestingly, we measured a very low, but detectable level of *CrFTL3* transcripts in dry seeds, the only organ in which *CrFTL3* expression was recorded.

Table 2 shows a summary of distinct organ-specific expression of the *FT*/*TFL1* genes. The highest levels were achieved by the *TFL1* subfamily genes in roots and by *MFT* genes in seeds. The distinct organ-specific expression of the *CrFTL1*, *CrTFL1*, and *CrCEN* genes in above-ground parts of the plants is documented. The results obtained by RT qPCR were in good agreement with coverage values (FPKM) estimated from Illumina data (Table S5).

**Table 2:** Expression of the *FT/TFL1* genes in various *C. rubrum* organs

	Cotyledon	Hypocotyl	Leaf	Stem	Apical Part	Flower	Root	Seed
*CrMFT1*	0–50	0–50	0–50	0–50	0–50	0–50	1000–5000*^a^*	>5000
*CrMFT2*	0–50	0–50	0–50	0–50	0–50	0–50	150–500	>5000
*CrBFT*	0–50	0–50	50–150	0–50	0–50	0–50	1000–5000	0–50
*CrCEN*	0–50	0–50	0–50	0–50	500–1000	0–50	1000–5000	0–50
*CrTFL1*	0–50	1000–5000	0–50	1000–5000	150–500	50–150	1000–5000	0–50
*CrFTL1**^b^*	>5000	0–50	>5000	0–50	50–150	0–50	0–50	0–50
*CrFTL2*	500–1000	500–1000	500–1000	500–1000	500–1000	500–1000	500–1000	150–500
*CrFTL3*	0–50	0–50	0–50	0–50	0–50	0–50	0–50	50–150

a Six classes of relative expression relative to actin were estimated by RT qPCR: >5000; 1000–5000; 500–1000; 150–500; 50–150; 0–50. Seedlings were cultivated in a growth chamber, adult plants grew in a greenhouse or in a growth chamber. Each estimation is based on 10–20 individuals.

b High expression only in plants induced to flowering.

Gene expression profiles provide valuable clues about possible function but more direct evidence is needed to elucidate gene functions with certainty. The functional proof may be achieved by a stable transformation of the plant with particular genes. Thus, we tried to transform *C. rubrum* by the floral dipping method described by Veit *et al.* (2006) using the same binary vector pFGC5941 (File S2). However, we failed to confirm DNA transfer by PCR. Moreover, we noticed that some control plants were able to grow on the plate with 5 mg l^-1^ of herbicide BASTA. Increased herbicide concentration killed all plants. Naturally resistant seedlings often germinated from large seeds produced by young plants. We concluded that the floral dipping method described by Veit *et al.* (2006) was not a reliable tool to achieve stable transformation of *C. rubrum*.

## Discussion

### CrFTL1 is a single floral activator of the FT/TFL1 gene family in C. rubrum

Flowering may be induced by environmental clues only when the plants are ready to bloom. Trees start to flower at the age of several years, sometimes even decades, in contrast with annual plants. Short-day *C. rubrum* represents an extreme even among annuals owing to its capability to flower at the seedling stage. Very early flowering and fast generation cycling is widespread among weedy plants, but it has been only rarely investigated at molecular level. *Ipomoea nil* (Japanese morning glory) is another example in which flowering can be induced as a seedling in short days (Hayama *et al.* 2007; Wada *et al.* 2010).

We generated a reference transcriptome of *C. rubrum* based on a superassembly combined from several individual assemblies by EvidentialGene pipeline and used it for a comprehensive survey of the *FT*/*TFL1* genes. Unlike *A. thaliana* or Japanese morning glory with two floral activators, *C. rubrum* relies on the single inducer from the *FT*/*TFL1* gene family – *CrFTL1*. The other two *FTL* paralogs – *CrFTL2* (Cháb *et al.* 2008) and the newly identified gene *CrFTL3* – were not involved in the control of flowering as documented by the constitutive or nearly zero transcription, respectively (Table 2), which was unchanged by floral induction.

### The structural evolution of the CrFTL2 gene indicates a functional shift

The *CrFTL2* gene has undergone a dramatic structural evolution and shown an accelerated substitution rate since its divergence from the sugar beet floral repressor *BvFT1* (Pin *et al.* 2010). A new exon and a long intron replaced the first 50 bp of the coding sequence. The novel exon encoded an α helix similar to the original chain, preserving the protein 3D structure (Figure 2B), despite a distinct primary sequence. The new intronic sequence was partly derived from the *CTL* gene, which is interesting in light of the recent finding that *A. thaliana* FT protein binds phosphatidylcholine *in vitro* (Nakamura *et al.* 2014). The novel intron is spliced out and the *CTL* sequence does not appear in a mature transcript. However, the recombination event, which probably restructured the gene, might have been a part of broader rearrangements affecting the control of both choline metabolism and flowering.

The spinach *SoFTL2* gene possesses the standard first exon similar to sugar beet *BvFT1*. Thus, the rearrangement affecting the *CrFTL2* gene (Figure 2A) had to occur relatively recently, after the separation of spinach and *C. rubrum* ancestors. The second homeolog *CrFTL2* copy is not functional which raises the question of whether it was inactivated before or after the origin of tetraploid *C. rubrum* from its so far unknown diploid ancestors.

The function of the *CrFTL2* gene remains enigmatic, but it is certainly not related to flowering control. A reliable protocol for gene transfer in *C. rubrum* has not been developed yet. Our attempt to adopt floral dipping method (Veit *et al.* 2006) for the stable *C. rubrum* transformation failed, but other methods like VIGS (virus induced gene silencing) may be successful in the future to provide evidence about the *CrFTL2* function.

The loss of floral repressor may allow seedlings to flower immediately after germination, if days are shortening in the fall. It appears to be a useful adaptation to a weedy opportunistic life style of *C. rubrum*. The loss of function often results in pseudogenization. However, relatively high and stable expression and the preservation of the 3D structure of the CrFTL2 protein after a complex gene rearrangement indicate a functional shift.

### The CrFTL3 and CrMFT genes diverged early

We discovered a third *FTL* paralog in Amaranthaceae. It arose early in the dicot evolution, most likely on the base of Caryophyllales (Figure 1). We cannot decide whether this clade is restricted to Amaranthaceae. We may have found *FTL3* homologs only in spinach and sugar beet, because no other Caryophyllales genomic drafts were available. Transcriptomic records are more numerous, but they may miss *FTL3* sequences due to very low transcription. We detected *CrFTL3* expression in seeds by RT qPCR, albeit at a negligible level. The FTL3 proteins contained several synapomorphic amino acid substitutions (Figure 3) which suggested a substantial functional divergence. We speculate that this gene may be involved in embryo growth control, which is in line with its ancient origin.

The two *CrMFT* genes were highly expressed in seeds and downregulated after germination (Figure 6), which implicates their role in seed dormancy control, as in *A. thaliana* (Xi *et al.* 2010). However, unlike *A. thaliana* having only one *MFT* gene, *C. rubrum* retained two *MFT* paralogs. Their different expression in roots indicates their function in vegetative growth.

### The genes of the TFL1 subfamily in C. rubrum show diverse expression patterns

The *CrTFL1* gene displayed the most variable expression, highly responding to darkness when applied on 5-d-old seedlings. Whereas *CrTFL1* was strongly inhibited by dark in hypocotyls and retained a low activity the following day, it was highly upregulated in roots but only at night (Figure 5). The TFL1 and FT proteins are non-cell-autonomous in *A. thaliana*; they spread among tissues (Conti and Bradley 2007; Corbesier *et al.* 2007). It is therefore possible that the CrTFL1 protein migrates at light from hypocotyl to the apex in *C. rubrum* seedlings. It may maintain apical meristematic activity, support vegetative growth, and counteract the FT protein in a similar way as in *A. thaliana* (Hanano and Goto 2011). A sudden decrease in *CrTFL1* transcripts caused by dark may contribute to floral induction in *C. rubrum*. The role of *TFL1* in roots was little investigated. The most recent evidence based on whole genome-association mapping (Lachowiec *et al.* 2015) suggests that *TFL1* acts as a repressor of root growth in *A. thaliana* seedlings. The coincidence of short roots in dark-treated seedlings (Figure 4) and *CrTFL1* upregulation by dark in roots (Figure 5) indicate a similar inhibitory function in *C. rubrum*.

*FT* and *TFL1* regulate a shoot architecture in various plants. For example, the *SFT* and *SP* genes, orthologs of *FT* and *TFL1* in tomato, control branching pattern and leaf shape in an antagonistic way. Their ratio, rather than individual transcript levels, shape tomato compound leaves (Lifschitz *et al.* 2014). We observed *CrFTL1* expression in leaves, whereas *CrTFL1* was transcribed in stem and roots, not in leaves (Figure 5). Thus, *CrTFL1* does not seem to affect leaf shape.

*CrBFT* was transcribed mostly in seedling roots, less in mature plants. Its expression was downregulated by dark. The opposite effect of darkness on *CrBFT* and *CrTFL1* could reflect their antagonistic roles in root growth regulation. *CrCEN* expression was little influenced by light regime, being limited to shoot apical parts in seedlings (Figure 5). In general, the three members of the *TFL1* subfamily exhibited very distinct patterns of organ-specific expression (Table 2), suggesting their different roles in plant growth and reproduction.

### Future perspectives

*C. rubrum* emerges as a very promising model for the studies of flowering owing to the absence of a juvenile phase and the presence of the single *FT*-like floral activator *CrFTL1*, which is upregulated 6 hr after lights-on, regardless of photoperiod (Drabešová *et al.* 2014). A midnight break inhibits both flowering and *CrFTL1* transcription (Cháb *et al.* 2008). The determination of the time period critical for floral induction and the availability of the reference transcriptome make it possible to conduct experiments to identify the genes controlling the induction to flowering in *C. rubrum*. The comparison of floral regulatory pathways among *C. rubrum* and other amaranths will improve our understanding of the adaptation of crops and weedy species to the ever changing environment.

## 

## Supplementary Material

Supplemental Material
