# Supplementary material for: The Evolution of the FT/TFL1 Genes in Amaranthaceae and Their Expression Patterns in the Course of Vegetative Growth and Flowering in Chenopodium rubrum
Source: G3 (Bethesda). 2016 Jul 28;6(10):3065–76. doi: 10.1534/g3.116.028639 (PMC5068931; doi:10.1534/g3.116.028639)
Supplement: Supplemental Material [file supp_6_10_3065__index.html]

The Evolution of the FT/TFL1 Genes in Amaranthaceae and Their Expression Patterns in the Course of Vegetative Growth and Flowering in Chenopodium rubrum — Supplemental Material 

# The Evolution of the *FT/TFL1* Genes in Amaranthaceae and Their Expression Patterns in the Course of Vegetative Growth and Flowering in *Chenopodium rubrum*

## Supplemental Material for Drabešova, *et al*, 2016

**Files in this Data Supplement:**

- File S1 - Cultivation of *C. rubrum* plants under various light regimes. (.pdf, 13 KB)
- Table S4 - The amino acid positions in FTL3 proteins different from other FT subfamily members. (.pdf, 54 KB)
- Table S5 - Coverage values (FPKM) of the *FT/TFL1* gene family members estimated in the Illumina transcriptomes from plants and organs of various ages (in days), cultivated under permanent light or induced to flowering by a single 12 h-period of darkness. (.pdf, 52 KB)
- File S2 - Cultivation of *C. rubrum* plants under various light regimes. (.pdf, 13 KB)
- File S3 - Diurnal rhythms of *CrFTL1* and *CrTFL1* expression in *C. rubrum* seedlings. (.pdf, 13 KB)
- Figure S1 - The primers used to sequence the *CrFTL* genes in *C. rubrum*. (.pdf, 173 KB)
- Figure S2 - Rhythmic expression of the *CrFTL1*, *CrTFL1* and *CrCAB1* genes under 12h light/12 h dark (left) and 6h light/18h dark (right). Gene expression under permanent light is shown by the grey curve. Double peaks of *CrCAB1* transcript levels suggest the regulation by endogenous clock and light. *CrFTL1* always peaks 6 h after light on. *CrTFL1* expression is very low, oscillates in opposite phase as *CrCAB1*. The time in hours is given on the x axis. (.pdf, 141 KB)
- Figure S3 - Relative expression of the *CrMFT1* and *CrMFT2* genes in various organs of *C. rubrum* plants under three light regimes. (.pdf, 121 KB)
- Table S1 - List of primers used in this study. (.pdf, 12 KB)
- Table S2 - The identity numbers (GB acc. numbers or genomic scaffold numbers) of the sequences used in phylogenetic analyses. (.pdf, 11 KB)
- Table S3 - The *FTL* genes in *C. rubrum* and spinach. (.pdf, 51 KB)
