# Supplementary material for: The Evolution of the FT/TFL1 Genes in Amaranthaceae and Their Expression Patterns in the Course of Vegetative Growth and Flowering in Chenopodium rubrum
Source: G3 (Bethesda). 2016 Jul 28;6(10):3065–76. doi: 10.1534/g3.116.028639 (PMC5068931; doi:10.1534/g3.116.028639)
Supplement: Supplemental Material [file supp_g3.116.028639_TableS4.pdf]

Table S4 The amino acid positions in FTL3 proteins different from other FT subfamily members. The residues responsible for the floral promoting activity of FT are also shown and marked by asterisk *. Two positions differentiating between the FTl and MFT subfamilies are also shown (**). Ratio numbers are derived from the alignment of 19 FTL, 15 MFT and 19 TFL1 proteins. The site numbers correspond to the A. thaliana FT protein.

Site	**Amino Acid CrFTL3/ SoFTL3/ BvFTL3**	Amino Acid FT other	Ratio	Amino Acid Smo MFT	Amino Acid Amborella MFT/ MFT1	Amino Acid MFT other	Ratio	Amino Acid TFL1	Ratio
7	**S/S/S**	P/A/H	14/1/1	P	P/P	P	11	P	19
12	**Q/E/Q**	R/H/S/G	13/1/1/1	R	K/K	R/K	9/2	R/K/G	17/1/1
34**	**R/R/R**	R/K	15/1	K	K/K	K	12	K/R/T	16/2/1
39	**A/A/A**	G/S	15/1	G	G/G	G	12	G	19
53	**I/I/I**	V	16	V	F/V	V/L/F	8/3/1	V	19
56	**Q/Q/L**	G	16	G	A/S	S/G/A/F/L	7/2/1/1/1	G/Q/H/S/N/L/E/F	6/3/2/2/2/2/1/1
78	**G/G/G**	S	16	S	S/S	S/H	11/1	S	19
82	**E/E/E**	L/Q/K	13/2/1	M	M/M	M/W	11/1	L/Q/M/S	16/1/1/1
**85***	**Y/Y/Y**	**Y**	16	W	W/W	W/Y/L	8/2/2	H/Y	18/1
92	**N/N/N**	D	16	D	N/D	D/N	9/3	D/N	18/1
97	**G/G/G**	T/A/S	14/1/1	A	S/S	T/M/A/S/G/L	4/3/2/1/1/1	T	19
**109***	**E/E/E**	**E**	16	I	I/M	M/V/T	8/2/2	E	19
**134***	**N/S/S**	**Y**/F/N	14/1/1	L	A/A	V/W/K/P/A/G/E/Q	3/3/1/1/1/1/1/1	R/V/S/N/H/G/A/T/F	4/4/3/2/2/1/1/1/1
**137***	**G/G/G**	**G**/E/S/Q	13/1/1/1	L	S/E	D/G/Q/A/I/N/P/H/S	2/2/2/1/1/1/1/1/1	S/T/G/I/W/E	11/2/2/1/1/1
**138***	**W/W/W**	**W**/M/Q	14/1/1	M	T/A	A/S/T/D/E/G	4/3/2/1/1/1	S/T/N/G/F/Y	12/2/2/1/1/1
**140***	**P/Q/Q**	**Q**/I/P/H	13/1/1/1	N	A/N	A/N/G	7/4/1	D/E	17/2
160	**T/S/M**	V/S	14/1	V	V/V	V/L/M	10/1/1	V/A	15/3
163	**T/T/T**	N	16	N	N/N	N/T	11/1	N	19
**166****	**R/R/R**	**R**	15	K	K/K	K	12	R	19
170	**Y/Y/Y**	S/C/T	9/5/1	S	N/S	N/S/A/V/K	6/2/1/1/1	A/P	18/1
171	**R/R/R**	G	15	R	K/R	K/R/H	7/3/1	**R**	19
