# Supplementary material for: The Evolution of the FT/TFL1 Genes in Amaranthaceae and Their Expression Patterns in the Course of Vegetative Growth and Flowering in Chenopodium rubrum
Source: G3 (Bethesda). 2016 Jul 28;6(10):3065–76. doi: 10.1534/g3.116.028639 (PMC5068931; doi:10.1534/g3.116.028639)
Supplement: Supplemental Material [file supp_g3.116.028639_TableS5.pdf]

**Table S5.** Coverage values (FPKM) of the *FT/TFL1* gene family members estimated in the Illumina transcriptomes from plants and organs of various ages (in days), cultivated under permanent light or induced to flowering by a single 12 h-period of darkness. Samples were taken 2h or 6 h after the end of the inductive dark period in 5 day old seedlings . Plants older than 12 days were transferred from permanent light to the greenhouse and cultivated under a natural photoperiod. The average values from two independent biological relicates were calculated.

| | 5 D 2 hours light | 5 D 6 hours light | 5 D permanent light | 12 Dinduced | 12 D permanent light | 25 D apical part | 32 D flower | 32 D young leaf | 32 D root |
|---|---|---|---|---|---|---|---|---|---|
| ***CrBFT*** | **1,52** | **3,96** | **2,88** | 0,57 | 0,52 | 0 | 0,99 | 0,06 | **4,63** |
| ***CrCEN*** | 0,2 | 0,1 | 0,33 | 0,71 | 0,87 | **2,26** | **4,3** | 0,9 | **27,6** |
| ***CrTFL1*** | **3,75** | **2,91** | **2,02** | 0,4 | 0,31 | 0,52 | 0,18 | 0 | **3,09** |
| ***CrFTL1*** | **11,8** | **24,63** | 0,15 | 0,25 | 0,43 | 0,38 | 0,69 | **1,58** | 0,27 |
| ***CrFTL2*** | **45,3** | **31,26** | **27,06** | **28,17** | **23,45** | **21,21** | **29,48** | **29,4** | **23** |
| ***CrMFT1*** | 0,47 | 0,53 | 0,36 | 0 | 0,07 | 0,07 | 0,49 | 0 | **8,61** |
| ***CrMFT2*** | 0 | 0,32 | 0,19 | 0,13 | 0,05 | 0,26 | 0,27 | 0,33 | **2,28** |
