# Supplementary material for: The Evolution of the FT/TFL1 Genes in Amaranthaceae and Their Expression Patterns in the Course of Vegetative Growth and Flowering in Chenopodium rubrum
Source: G3 (Bethesda). 2016 Jul 28;6(10):3065–76. doi: 10.1534/g3.116.028639 (PMC5068931; doi:10.1534/g3.116.028639)
Supplement: Supplemental Material [file supp_g3.116.028639_FileS2.pdf]

File S2. Cultivation of *C. rubrum* plants under various light regimes.

Experimental plants were germinated and cultivated in the growth chamber (Percival Scientific E-36L) at 20 °C and relative humidity 60 % until the age of 15 days. Three consecutive dark periods lasting 12 h or 18 h were applied to 5 day-old seedlings. Control plants were kept under constant light. Aerial parts of seedlings were collected each 3 hours. Sampling in dark was performed under a dim green light (about 520 nm, Green Hornet LED headlight). To estimate flower development, 15 individuals from each experimental treatment were kept intact and grown under constant light until the age of 14 days. Morphological changes at the shoot apex associated with flowering were observed under a stereomicroscope at magnification 4x (Motic Deutschland, Germany).

15 day-old plants were transferred to 8 x 8 cm pots with soil substrate and cultivated in a greenhouse under a semi-natural photoperiod (about 16 h light/8 h darkness) or in the growth chamber with constant light for an additional four weeks (mature plants).

We applied three photoperiodic regimes to growing plants: 12h light/12 h dark since the from of 3 days onwards, three periods of 12h light/12 h dark followed by permanent light, or constant light during the entire experiment. Seeds of *C. rubrum* were germinated and cultivated as described above. We replaced the Hoagland solution each second day and cleaned all ELISA plates. The first 12 h-dark period was applied on 5 day-old seedlings, whereas control plants were kept under constant light. The first samples for RNA extraction were taken 6 h after lights-off, during the third dark period. Seedlings were cut into four parts - cotyledons, root, hypocotyl and apical part including apical meristem and adjacent tissues (size about 1 mm). The tissues from 5 individuals were combined and frozen in liquid nitrogen, in two replicates. The second sampling occurred 6 h after the end of the third dark period, the tissues were collected as described above. Plants from the second light regime were transferred to permanent light after the third dark period. The third sampling took place after 5 days at noon, when plantlets were 10 days old. Leaf samples consisted of the first true leaves and hypocotyls were replaced by stem internodes. The fourth and further samplings occurred after each additional 5 days. 20 day-old plants were transferred to Jiffy-7 (Jiffy Group, Norway) discs, 25 day-old plants were transplanted to autoclaved soil substrate filled in 8 x 8 cm pots. Because this manipulation might have influenced gene expression particularly in roots, we examined only growth habitat in 32 day-old plants.
