# Supplementary material for: The Evolution of the FT/TFL1 Genes in Amaranthaceae and Their Expression Patterns in the Course of Vegetative Growth and Flowering in Chenopodium rubrum
Source: G3 (Bethesda). 2016 Jul 28;6(10):3065–76. doi: 10.1534/g3.116.028639 (PMC5068931; doi:10.1534/g3.116.028639)
Supplement: Supplemental Material [file supp_g3.116.028639_FileS3.pdf]

File S3. Diurnal rhythms of *CrFTL1* and *CrTFL1* expression in *C. rubrum* seedlings

We monitored rhythmic *CrFTL1* expression in aerial parts of seedlings under two photoperiods, 12 h and 6h, during 72 h. Light-grown seedlings were subjected to the first dark period at an age of 5 days (time 0). *CrFTL1* peaked 6 h after the light onset under inductive 12h L - 12h D conditions which resulted in 100% rate of flowering after two weeks, as previously documented (Drabešová et al. 2014). The comparison between the rhythmic patterns of *CrCAB1*, a homolog of the well-studied *CAB* gene regulated by endogenous clock and light in *A. thaliana* (Millar and Kay, 1996), and *CrFTL1* revealed a phase shift - *CrCAB1* maxima lagged 3 h behind the *CrFTL1* peaks (**Fig** A). A minor secondary peak and shoulder were observed at the end of the dark periods at 36 h and 60 h, respectively, in *CrCAB1* rhythms. They may be interpreted as the response to two signals: light and endogenous clock. A small decrease in *CrCAB1* transcript levels after the second dark period at 39 h may be explained by the combination of the activation by light and the inhibitory signal from the endogenous clock, as suggested by free running rhythms estimated under permanent light. A shoulder rather than a peak occurred at 60 h, most likely due to the clock entrainment. The *CrFTL1* rhythms were smooth, without double peaks or shoulders.

Under a short 6 h photoperiod, less permissive for flowering (75%), the *CrFTL1* maxima were about 4 times lower then under 12 h photoperiod. They occurred 6 h after lights-on (**Fig B).** The rhythms of *CrCAB1* and *CrFTL1* were in the same phase under this photoperiodic regime. Thus, phase shift between the *CrCAB1* and *CrFTL1* rhythms varied depending on the photoperiod – 3h shift in 12 h photoperiod and 0 shift in 6 h photoperiod. In contrast, *CrFTL1* peaked 6 h after light onset under both regimes. This suggests that light signal rather than endogenous clock activates *CrFTL1* expression.

The *CrTFL1* transcript levels exhibited rhythmicity, although with a very low amplitude. The maxima opposed the *CrCAB1* peaks, double peaks and shoulders were observed under both photoperiodic regimes (**Fig A, B**). This observation indicates that *CrTFL1* transcript levels in seedlings were regulated similarly to *CrCAB1*, but in the opposite direction. The *CrCEN* and *CrBFT* transcription in aerial parts of seedlings was too low to allow a reliable detection by RT qPCR.

References

Millar AJ, Kay SA. 1996. Integration of circadian and phototransduction pathways in the network controlling *CAB* gene transcription in Arabidopsis. *Proceedings of the National Academy of Sciences of the USA* 93(26): 15491−15496.
