# Supplementary figures and images for: The Evolution of the FT/TFL1 Genes in Amaranthaceae and Their Expression Patterns in the Course of Vegetative Growth and Flowering in Chenopodium rubrum
Source: G3 (Bethesda). 2016 Jul 28;6(10):3065–76. doi: 10.1534/g3.116.028639 (PMC5068931; doi:10.1534/g3.116.028639)

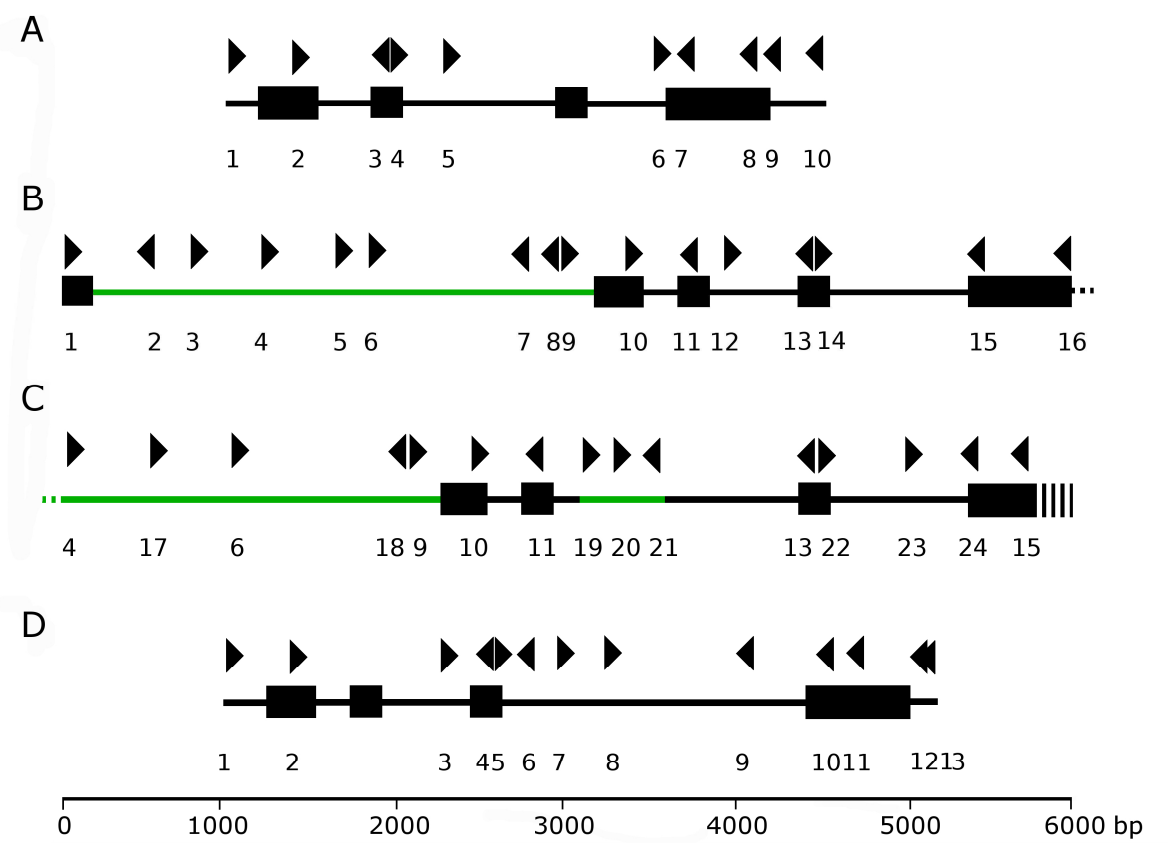

**Figure S1** The primers used to sequence the *CrFTL* genes in *C. rubrum*.

Supplement: Supplemental Material [file supp_g3.116.028639_FigureS1.pdf]

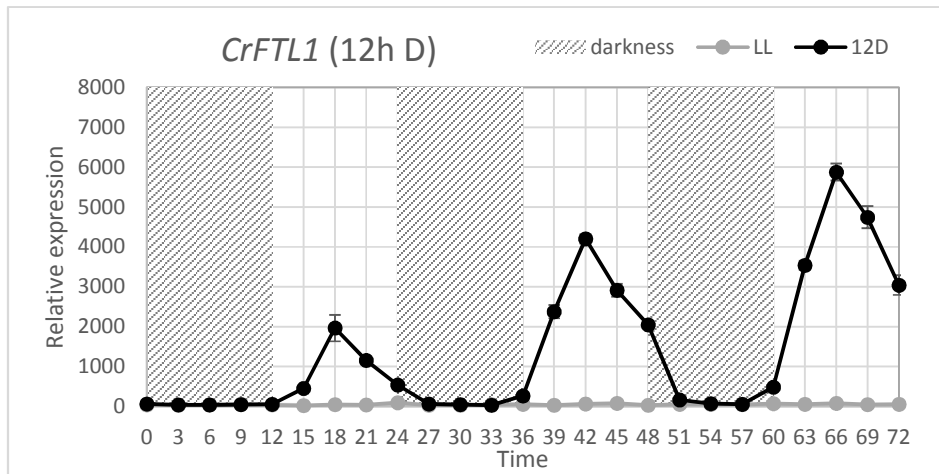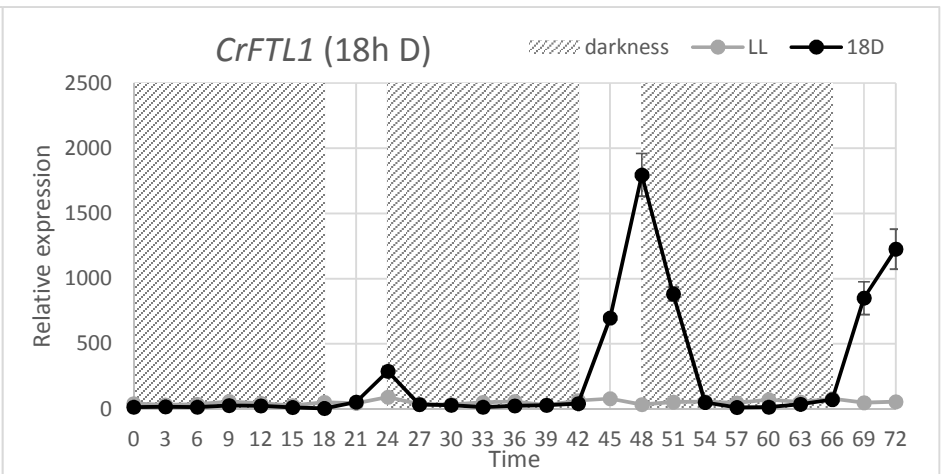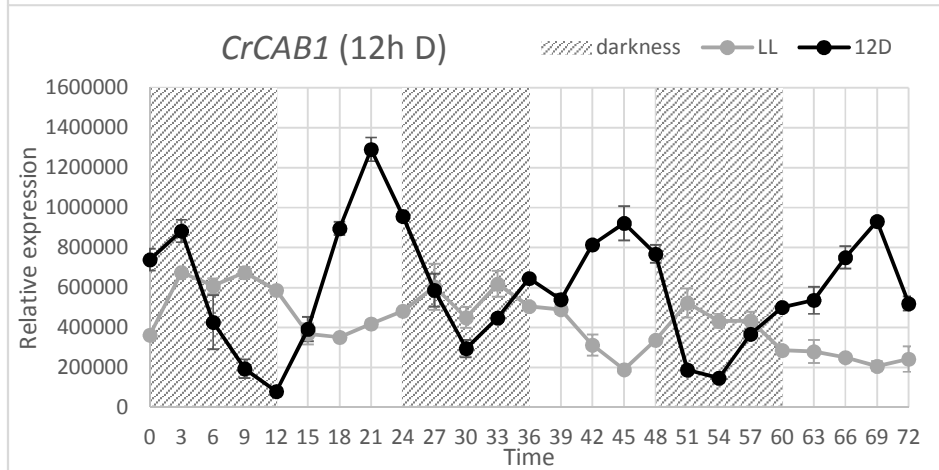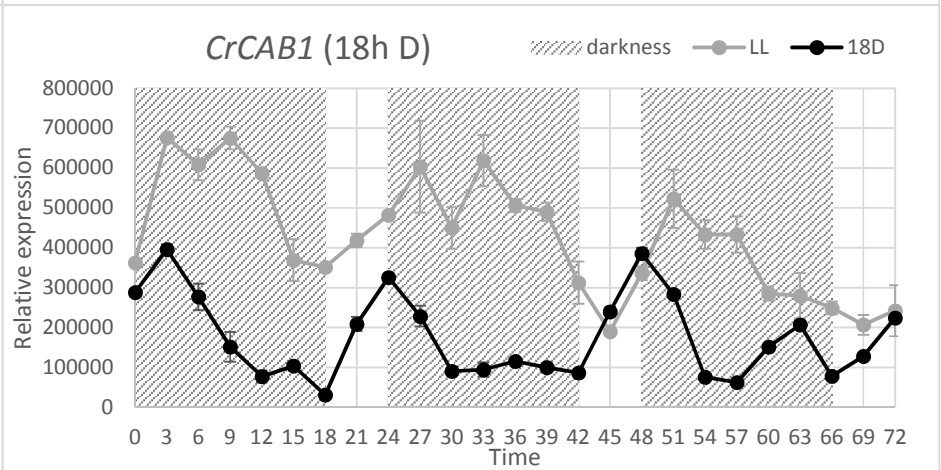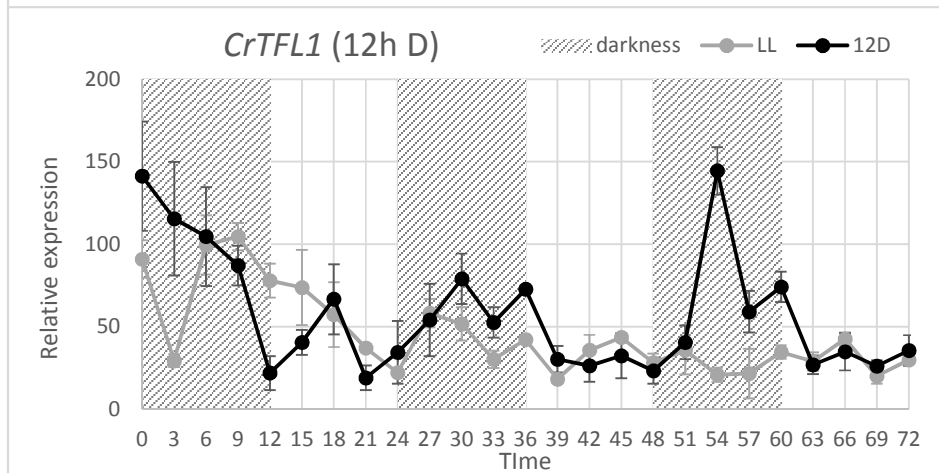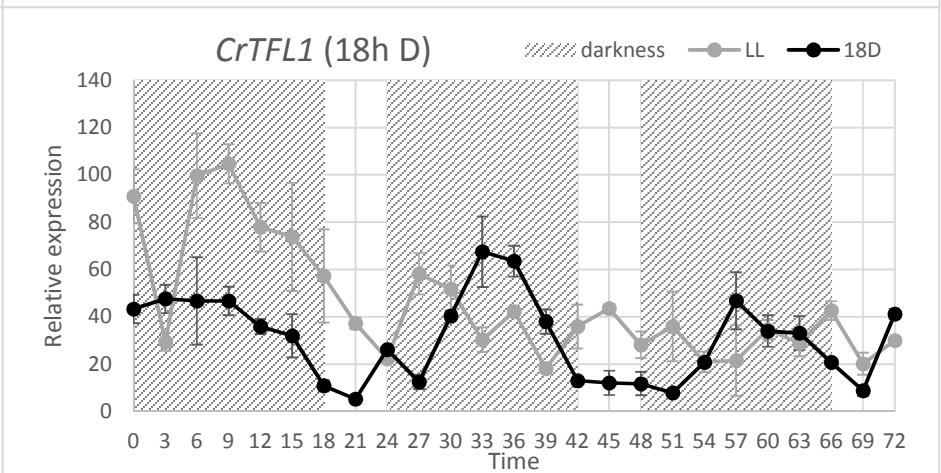

Supplement: Supplemental Material [file supp_g3.116.028639_FigureS2.pdf]
