# Supplementary material for: The Evolution of the FT/TFL1 Genes in Amaranthaceae and Their Expression Patterns in the Course of Vegetative Growth and Flowering in Chenopodium rubrum
Source: G3 (Bethesda). 2016 Jul 28;6(10):3065–76. doi: 10.1534/g3.116.028639 (PMC5068931; doi:10.1534/g3.116.028639)
Supplement: Supplemental Material [file supp_g3.116.028639_FigureS3.pdf]

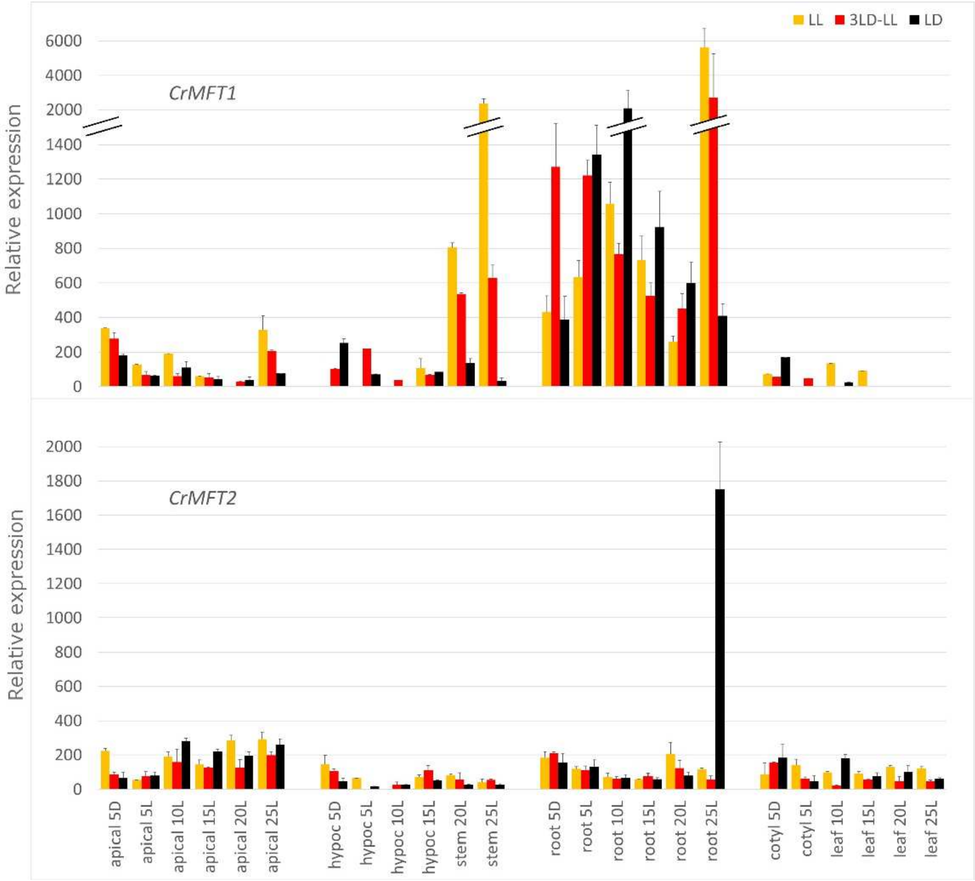

Figure S3. Relative expression of the *CrMFT1* and *CrMFT2* genes in various organs of *C. rubrum* plants under three light regimes.

Relative expression of the *CrMFT1* and *CrMFT2* genes in various organs of *C. rubrum* plants under three light regimes. LL - permanent light (yellow), 3 LD –LL - three consecutive periods 12 h light/ 12 h dark followed by permanent light (red), LD - 12 h light/ 12 h dark (black).
