# Supplementary material for: The Evolution of the FT/TFL1 Genes in Amaranthaceae and Their Expression Patterns in the Course of Vegetative Growth and Flowering in Chenopodium rubrum
Source: G3 (Bethesda). 2016 Jul 28;6(10):3065–76. doi: 10.1534/g3.116.028639 (PMC5068931; doi:10.1534/g3.116.028639)
Supplement: Supplemental Material [file supp_g3.116.028639_TableS1.pdf]

Table S1. List of primers used in this study

FTL1 primers (a)	
1	TAACCAGGCCTCTCAGCTTC
2	AATTCAGGCCCTCTCAGGTT
3	GCCCAAGTAATCCACACCTG
4	TGCAAGTACTCCCTCAGGTG
5	TAATATCCCTTTCAAGAATCGAA
6	TTTGGCCAAGAGTTGTTTGG
7	CACCGATGGTCTTGGACTCT
8	CCCTCCCTTTGACAATTGAA
9	TCCAATGATCTCTTCACGGTAG
10	GGTACTTATTTTAGAAAAGTGTTGAGC

FTL3 primers (d)	
1	CAACTGCTATAAAAACCCCAAAA
2	CAACCCACCAATGATTGAAA
3	ATGTGACTCGACAAGTGTGAA
4	CCCAAACTGATGCCTCCAGT
5	ACTGGAGGCATCAGTTTGGG
6	ATAGCCGCAAAGCAACTTGC
7	TACACTGCTGCCATTTGTGG
8	TTTTTCCCTGTTACCGCAGT
9	TGCATGTCTTCTTAGCAAAGGA
10	TGCCTTTCCGAGTGACGAA
11	GCAACAGGCAAGCCAAGATT
12	CTCCAAGCAAGGATTGAAGC
13	ATGATGCTCCAAGCAAGGAT

FTL2 primers (b,c)	
1	AATGAGTTCCGGTTGCAGTC
2	AGGGGAAAGGGCCATGATTG
3	ACCCCACTTTCTCTTCTTGTGT
4	GGATAGTTGGGTGGGTGCTT
5	GGTGGTTGCTGTTGTGCATT
6	TGAAGTGTTGGCTGTTGAGA
7	CCACATGAAGTCCAAGAAGACAC
8	GCATGAAGGGTGGTGTGAGA
9	ACACCACCCTTCATGCAGTA
10	CTAGGGTCGAGGTTGGAGGT
11	GAGCATCAGGATCCACCATT
12	GCTCCCTCTATTTCTGCTTCAA
13	GAGGTCCCGGGAATATCAGT
14	ATATTCCCGGGACCTCTGAT
15	TCCTTCCCTTCAACAATTGAA
16	TGCCCACTTTGTAACTTTGTG
17	TGTGGTATGAGTTGATTTAGGTGAA
18	GCATGAAGGGTGGTGTGAGA
19	AGAAAATGCCACATATCAGAGGA
20	ATAGCTAAGCCGCAAGGTGA
21	TTTGGCCTCTCGTATTTTCG
22	AGGGATTACTGGTGCATCC
23	CCACCTGCACCAATAGTTGT
24	TACGTCCTCTTGGCCAAAACA

qPCR primers		complete cDNA sequence primers	
actin		***CrTFL1***	
ACT-2_for	CATCGTGCTCAGTGGTGGTA	*CrTFL1_COMPL_F3*	ACATGGCAAGGATGATAGAACC
ACT-2_rev	TGAGGGATGCAGGGATAGAC	*CrTFL1_COMPL_R3*	AGCAGCAGTTTCTCTTTGAGC
CrFTL1		***CrMFT1***	
CrFT720-52For	TGCACTGGTTGGTGACTGAT	*CrMFT1_COMPL_F1*	ATTGATCCCTTAGTCGTCGGA
CrFT720-298Rev	CCCTCCCTTTGACAATTGAA	*CrMFT1_COMPL_R2*	TCCACATCAAAACGTACAACCC
CrFTL2		***CrMFT2***	
CrFT 787-361For	ATATTCCCGGGACCTCTGAT	*CrMFT2_COMPL_F1*	GGCACACATACACTCACAGTTTA
CrFT 787-536Rev	TTCAGCGAAGGCTTCTGTTT	*CrMFT2_COMPL_R1*	TCGTTTCTTATTAGCAGGCTCT
CrFTL3		***CrCEN1***	
FTL3_F12	GGCGTCCAAATTTCTTCACC	*CrCEN_COMPL_F1*	CTCTTCCAAATCAAACCCCGT
FTL3_R12	TCAACGTCTCCTTCCTCGAT	*CrCEN_COMPL_R1*	TGGCTATAAGTCCGACTCAAGA
CrTFL1		***CrBFT***	
qTFL1_F7	TCAGTCTCCTCCAAACCTCG	*CrBFT_COMPL_F1*	TTCAGTTTCCATGGCAAGAGG
qTFL1_R7	ATCACTTGGTCCAGGCACAT	*CrBFT_COMPL_R1*	TCTTGCTTAGGGAAAATACTTGC
CrMFT1			
CrMFT1_qF1	ATACATGGGACCAAAACCGC		
CrMFT1_qR1	AAGTAAACTGCAGCAACCGG		
CrMFT2			
CrMFT2_qF1	CAAAAGCAGCAGTTGGGGAT		
CrMFT2_qR1	CAGGCTCTTTCTGGGCATTG		
CrCEN1			
CrCEN_qF2	TTCCTGGACCTAGTGACCCTT		
CrCEN_qR2	CTGTGTATGCCGATGTTTGGC		
CrCAB1			
CrCAB1_F1	ATTTCCCGCTCGAACAAACC		
CrCAB1_R1	AGCGGTCTTCCTCATTGTGA		
CrBFT			
qBFT_F1	GGTAGACCCAGATGCTCCAA		
qBFT_R1	CCTCCCTCTTGTTTGCTTGA		
