# Supplementary material for: The Evolution of the FT/TFL1 Genes in Amaranthaceae and Their Expression Patterns in the Course of Vegetative Growth and Flowering in Chenopodium rubrum
Source: G3 (Bethesda). 2016 Jul 28;6(10):3065–76. doi: 10.1534/g3.116.028639 (PMC5068931; doi:10.1534/g3.116.028639)
Supplement: Supplemental Material [file supp_g3.116.028639_TableS2.pdf]

Table S2. The identity numbers (GB acc. numbers or genomic scaffold numbers) of the sequences used in phylogenetic analyses.

	AtMFT	NM101672	
	AtBFT	NM_125597	
	ATC	NM_128315	
	AtFT	NM_105222	
	AtTSF	NM_118156	
	AtTFL1	ATU77674	
	AtriMFT	XM_006849682	
	AtriMFT1	XM_006841802	
	AtriCENlike	XM_006835987	
	AtriHd3a	XM_006858577	
	BvMFT	XM_010687957	
	BvMFTlike	HM448918	
	BvBFT	HM448916	
	BvCEN	HM448913	
	BvCENlike	XM_010685445	
	BvFT1	HM448910	
	BvFT2	HM448912	
	BvFTL3	Bvchr6.scaff028	0551.scaff01119
	CrMFT1	KU140602	
	CrMFT2	KU140603	
	CrBFT	KU140599	
	CrCEN	KU140601	
	CrFTL1	EU128013	
	CrFTL2	EF445636	
	CrTFL1	KU561656	
	CrCAB1	KU140600	
	JcMFT	NM_001306009	
	JcMFTlike	NM_001308696	
	JcFT	KF113881	
	JcTFL1a	NM_001306038	
	JcTFL1b	NM_001306004	
	JcTFL1c	NM_001308743	
	SmoMFT	XM_002990181	
	SoMFT1	scaff12981	
	SoMFT2	scaff10102	
	SoBFT	scaff14048	
	SoCEN1	scaff4602	
	SoCEN2	scaff14350	
	SoFTL1-1	scaff16133	
	SoFTL1-2a	scaff8759	
	SoFTL1-2b	scaff8759	
	SoFTL2	scaff39498	
	SoTFL1	scaff68707	scaff48765
	VvFT	DQ504308	
	ZmZCN9	EU241900	
	ZmZCN10	NM_001112778	
	ZmZCN11	NM_001112793	
	ZmZCN8	EU241924	
	ZmZCN15	EU241930	
	ZmZCN19	EU241934	
	ZmZCN2	EU241918	
	ZmZCN3	EU241919	
genomic	*CrFTL1*	KT992793	
genomic	*CrFTL2*	KT992794	
pseudo	*CrFTL2*	KU140604	
genomic	*CrFTL3*	KT992795	
