# Supplementary material for: The Evolution of the FT/TFL1 Genes in Amaranthaceae and Their Expression Patterns in the Course of Vegetative Growth and Flowering in Chenopodium rubrum
Source: G3 (Bethesda). 2016 Jul 28;6(10):3065–76. doi: 10.1534/g3.116.028639 (PMC5068931; doi:10.1534/g3.116.028639)
Supplement: Supplemental Material [file supp_g3.116.028639_TableS3.pdf]

Table S3. The *FTL* genes in *C. rubrum* and spinach. The lengths of introns and exons in bp. The scaffolds of the genomic draft Spinach 1.0.1 containing respective genes are given..

	**Exon1a**	**Intron 1a**	**Exon1**	**Intron 1**	**Exon2**	**Intron2**	**Exon3**	**Intron3**	**Exon4**	**Identification**
CrFTL1			>**201**	238	**62**	1212	**41**	708	**>224**	KT992793
SoFTL1-1			>**201**	136	**62**	1465	**41**	1266	**>224**	Scaffold 16133
SoFTL1-2a			>**201**	167	**62**	1119	**41**	3389	**>224**	Scaffold 8759
SoFTL1-2b			>**201**	150	**62**	963	**41**	2224	**>224**	Scaffold 8759
CrFTL2	**>50**	3157	**160**	139	**62**	792	**41**	1078	**>191**	KT992794
SoFTL2			**>216**	152	**62**	1454	**41**	85	**>224**	Scaffold 39498
CrFTL3			>**201**	108	**62**	903	**41**	2213	**>224**	KT992795
SoFTL3			**>213**	89	**62**	1918	**41**	1798	**>224**	Scaffold 30409
